# Complete stent coverage of the coronary bifurcation is an outdated concept

**DOI:** 10.1093/ehjcr/ytae168

**Published:** 2024-03-30

**Authors:** Pitt O Lim

**Affiliations:** Department of Cardiology, Atkinson-Morley Wing, St George’s University Hospitals Foundation Trust, Blackshaw Road, Tooting, London SW17 0QT, UK

Güner *et al.*^1^ succinctly reflect on two treatment strategies in critiquing Gupta *et al*.’s^2^ report, with respect to ostial left anterior descending (LAD) artery disease. Either nailing the LAD ostium with a stent (OS) or crossing the stent over (CS) into the left main (LM) coronary artery. The OS approach avoids covering the LM that increases procedural complexity; stenting an unobstructed LM and plaque-ploughingly jailing the circumflex artery, thus converting an otherwise simple lesion into a bifurcation conundrum. However, OS is ‘hit-and-miss’, flush ostial LAD stent landing is impossible, and distal LM is never disease free. From registry data, both methods have high rates of combined stent failure and death in excess of 10% within the year.^3^

The hybrid provisional stenting (HPS) strategy could be a middle way between two extremes.^4^ There is a need to cover any balloon-injured segment of coronary artery with a stent, including the bifurcation carina to reduce restenosis; the so-called *complete stent coverage philosophy*. There are manifold two-stent techniques; double-barrel, culotte, double-kiss crush, T-and-protrusion, etc. These are complicated multi-stepped procedures with heightened adverse outcomes, even for the skilled operators. On the other hand, the drug-coated balloon (DCB), which is as good as the stent in reducing restenosis but is superior in the presence of vasculitis, T-stenting an ostial coronary lesion is now feasible.^4^ In other words, the stent is placed shy of the ostium, missing it, and the uncovered ostium is treated with a DCB as exemplified in *Figure 1*.

**Figure 1:**
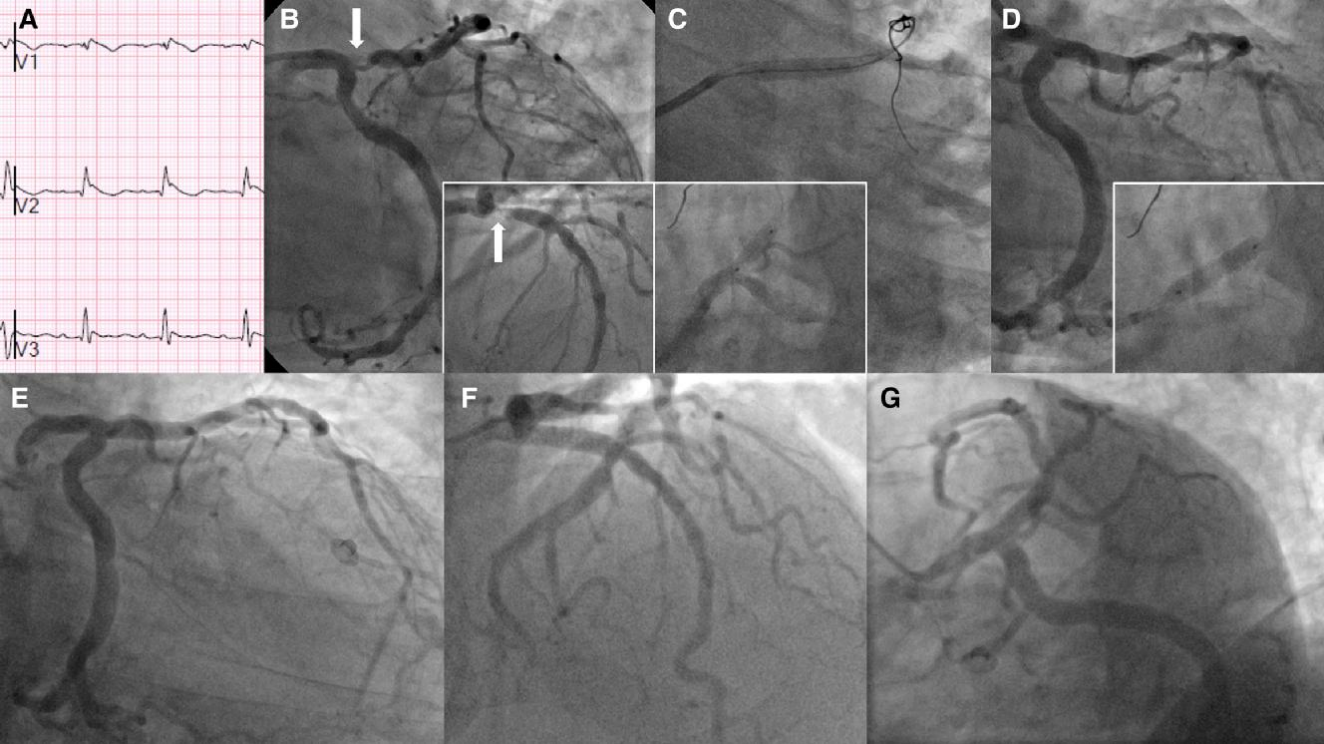
(*A*) Brugadoid-type ST elevation with a peak troponin of 450 (<14) ng/L. His right coronary artery is small and non-dominant, and it arises anomalously from the left coronary cusp with mid vessel disease (R-AAOCA) that could explain the electrocardiographic appearance. A subsequent ambulatory Holter monitoring did not pick up significant cardiac arrhythmias. (*B*) Critical left anterior descending (LAD) artery with Medina 0.1.0 ostial lesion on caudal radiographic view and the inset discloses the same disease in the cranial radiographic angulation. (*C*) The ostial LAD lesion was wire-cut with double-buddy—note the wires on top of the inflated balloon. The inset illustrates the proximal LAD stent deployment deliberately avoiding the LAD ostium. (*D*) The inset shows a DCB inflated in left main (LM) coronary artery and LAD, and the main picture reveals the final angiographic result. (*E*–*G*) Follow-up coronary angiogram at 6 months depicts the widely patent LM and LAD in right anterior oblique caudal, cranial and spider radiographic views respectively. The hyperdominant circumflex artery is not compromised.

When Andreas Grüntzig introduced coronary angioplasty in 1977, the balloon-dilated artery acutely occluded in 10% of cases, necessitating surgical rescue. These were mostly thrombotic occlusions, now abolished by modern antiplatelet therapy. Occlusive dissection is rare in current practice. For instance, it occurred in 1/1751 (0.06%) *de novo* DCB cases without stenting over 10 years, half of these patients had complex or calcific disease.^5^ Conversely, stent edge dissection (SED) is associated with 5% mortality in 3 months.^4^ The distal SED is more prognostically important because mechanistically, the dissection flaps are counterflow and extension occur with each heartbeat leading to stasis and stent thrombosis, compared with proximal SED that is sealed by antegrade blood flow.^4^ This explains why Gupta *et al.*’s^2^ LM proximal SED has a predictable good outcome. This brings us back to the HPS technique in *Figure 1*. This was an 87-year-old man with an acute coronary syndrome and hypokinesia in the LAD territory on echocardiogram. His coronary angiogram found critical ostial LAD disease. The lesion was wire-cut,^6^ the proximal LAD was stented, and the LM into the LAD was treated with a DCB. He had a restudy at 6 months that demonstrated widely patent LM and LAD. His cardiac function normalized, and he remains symptom-free at 4 years, aged 91. According to UK’s Office of National Statistics, an over 85-year-old man has an annual survival rate of about 83%, the 4-year accumulated mortality for this man would be 68%. Highlighting the need to minimize procedural risks where possible, especially in the elderly, and mixing stent with DCB will achieve this.

**Consent:** Obtained for the figure.

**Funding:** None declared.

## Data Availability

The data underlying this article are available upon reasonable request.
